# Effects of immunization program on morbidity and mortality rates ofvaccine-preventable diseases in Turkey

**DOI:** 10.3906/sag-2008-177

**Published:** 2020-12-17

**Authors:** Tufan NAYİR, Ersin NAZLICAN, Musa ŞAHİN, Fatih KARA, Emine ALP MEŞE

**Affiliations:** 1 Ministry of Health, Ankara Turkey; 2 Department of Public Health, Faculty of Medicine, Çukurova University, Adana Turkey

**Keywords:** Vaccination, vaccine-preventable diseases, communicable diseases, immunization

## Abstract

**Background/aim:**

This study aims to serve as a unique consolidated source in Turkey’s vaccination history and as an example for other countries by objectively revealing the change in mortality and morbidity rates in Turkey following the beginning of vaccination without asserting any claim on the benefits or risks of vaccines. This unbiased research will also help health professionals identify the challenges more easily when they face with the people who hesitate to vaccinate their children.

**Materials and methods:**

Descriptive research design is adopted in this study. The coverages of vaccinations, mortality, and morbidity rates were identified through a retrospective analysis of the data provided from the Ministry of Health of Turkey. The data provided by the Turkish Statistical Institute were used for the identification of the population by the year. Mortality and morbidity rates were calculated based on these data

**Results:**

Morbidity rates, mortality rates, and vaccine coverages are all presented in years. Successful interventions have been observed in the eradication of polio, the elimination of maternal neonatal tetanus, and also in combating with other diseases. A decline in pertussis mortality from 0.59 to 0.06 along with a decline in diphtheria morbidity under to 0.0001 were recognized; additionally the last death due to poliomyelitis was observed in 1998. Only 4 deaths occurred in the measles epidemic in 2013. With the initiation of vaccination, both the morbidity of the rubella with the ratio of 3.12/100,000 and the mortality of pumps with the ratio of 25/100,000 fell to zero. Also, no death due to neonatal tetanus has been recorded since 2014.

**Conclusion:**

The present study demonstrates that many possible diseases and deaths have been prevented through vaccination studies. In this regard, this study demonstrating the importance of vaccination presents that all individuals in the society have a responsibility in this scope when the communicable diseases and wars are taken into consideration. The main responsibility is to ensure that they and their children are vaccinated against communicable diseases that are risky for society’s health.

## 1. Introduction

It has been observed that the microorganisms led to radical changes in political and social structures in communities by causing outbreaks with the emergence of the communicable diseases such as plague, smallpox, typhus, typhoid, cholera, influenza, and malaria [1].

Today, an up-to-date vaccination schedule is established in Turkey with the gradual development of new vaccines, strains, and application methods.Vaccine schedules and vaccination coverages are constantly changing and improving thanks to the recent developments in technology. The Expanded Programme on Immunization (EPI) consisting of the rules to be observed for management of the nationwide and provincial immunization services in Turkey became effective as of 1981.The main purpose of the EPI is to vaccinate every newborn baby against diseases through the immunization schedule. The word “expanded” is used to emphasize that unvaccinated or incomplete vaccinated babies and children should be vaccinated as soon as they are detected and that this implementation must be maintained evenly everywhere throughout the country. Besides, EPI has many goals such as: reaching 95% vaccination coverages across the country, eliminating tetanus, neonatal tetanus as well as such diseases related to diphtheria, pertussis, hepatitis-b, tuberculosis, mumps, hemophilus influenza type b, and also controlling invasive pneumococcal diseases related to streptococcus pneumonia, maintaining vaccine safety, strengthening the notification system and ensuring the participation of the community. Three new agents (rubella, mumps, and hemophilus influenza type b) have been added to the vaccination schedule in EPI since 2006. Since 2008, the pentavalent vaccine including DaPT-IPV-Hib and the conjugated pneumococcal vaccine have been launched. Constantly supported by elimination and eradication programs and with the improvements in recording and notification systems, EPI is becoming more effective day by day [2,3].

On the other hand, the World Health Assembly aimed to eradicate poliomyelitis from all over the world until 2000. Surveillance and laboratory investigations have been initiated in Turkey towards acute flaccid paralysis cases under the age of 15 in compliance with the aim of eradication of poliomyelitis [4].

In Turkey, also pertussis and diphtheria case records are laboratory-supported and case-based surveillance systems [5].

Surveillance studies for viral hepatitis (Only Hepatitis-A and Hepatitis-B) are ongoing, but case-based data for these diseases have not been collected. Although endemicity continues for chickenpox and mumps throughout the country; no confirmed data has been collected, besides, no monitoring case-based and laboratory-supported data exist for these diseases [5].

In order to have a strong surveillance system on data, laboratory-supported definitive case surveillance has been started as of 2005. Including Measles, Rubella, and Congenital Rubella Syndrome Elimination Program, case-based and necessarily confirmed or epidemiologically related cases are considered as confirmed cases and specific surveillance is carried out based on WHO standards.

Today, campaigns against vaccines started to be conducted through social media or other mass media platforms. As a result of these campaigns, the debates on the harm of vaccines have increased and vaccine hesitancy and refusal have consequently scaled up all over the world and took place in the WHO’s list of 10 threats to global health in 2019. Therefore, this brief report was written with the aim of being a collective single source in regards to the history of vaccination in the country, providing an example to other countries, and supporting the health professionals to raise awareness of the parents with vaccine hesitancy for their children having possible risks while taking the parents’ decisions on vaccination. This study presents the figures on mortality and morbidity rates seen before the implementation of vaccination in Turkey and also the changes in those figures after the vaccination practices are imposed in Turkey as a result of the gradual developments. The results aim to impartially demonstrate the difference which may affect the decision of the people who have vaccine hesitancy without clearly claiming the benefits of vaccination.

## 2. Materials and methods

The number of mortality and morbidity was identified through a retrospective analysis of the archival records provided from the Ministry of Health of Turkey. In the records of the Ministry of Health, mortality rates were calculated for 1.000.000, while it was 100,000 for morbitiy rates. Mortality and morbidity rates based on these data and vaccination coverages based on WHO data World Health Organisation (2020). Spreadsheet of the vaccinaton of countries [online]. Website https://apps.who.int/immunization_monitoring/globalsummary/ timeseries/ tscoveragedtp3.html [accessed 10.09.2020] were compared in figures. The data of Turkish Statistical Institute Turkish Statistical Institute (2019). The Population Statistics by years, age group and sex 1935-2019 document [online]. Website u294d Tablo.do?istab_id=1588 [accessed 10.09.2020] were used for the identification of the population by years.

**Figure:**
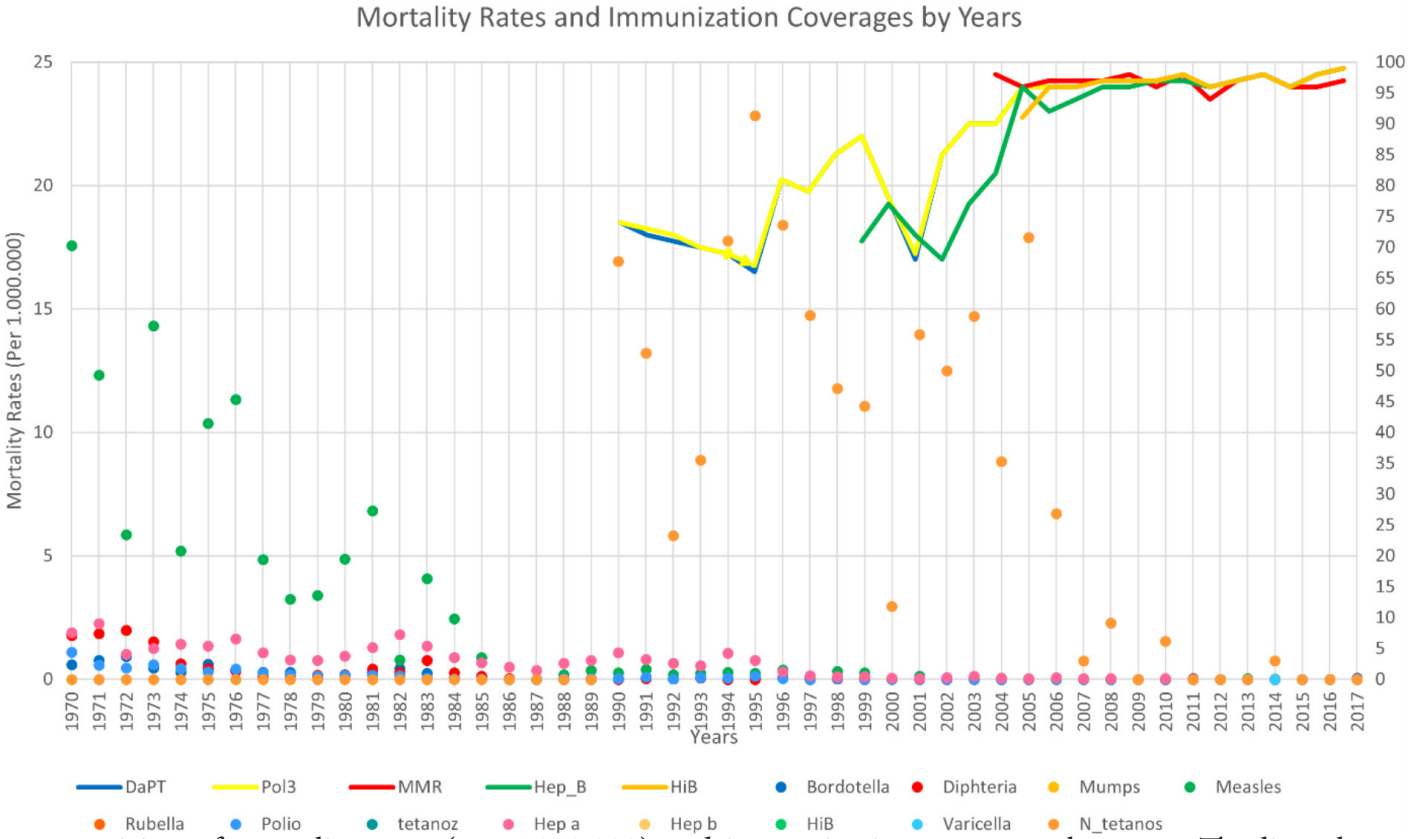
The comparision of mortality rates (per 1.000.000) and immunization coverages by years. The line chart shows vaccine coverages, and as it is shown with numbers on the right side, it increases from 0 to 100 from 1970 to 2020. As the immunization records started at 1990, the lines start at 1990. DaPT line represents Diphteria, Pertussiss, and Tetanus; MMR line represents Mumbs, Measles, and Rubella; Pol3 represents Polio; Hep_B represents Hepatitis B; Hib represents H.influenza type b. The dot plot in the figure represents mortality rates. As it can be seen by numbers on the left side, the mortality rate increases from 0 to 25 (per 1.000.000), when the timeline goes from 1970 to 2020.

**Figure 2:**
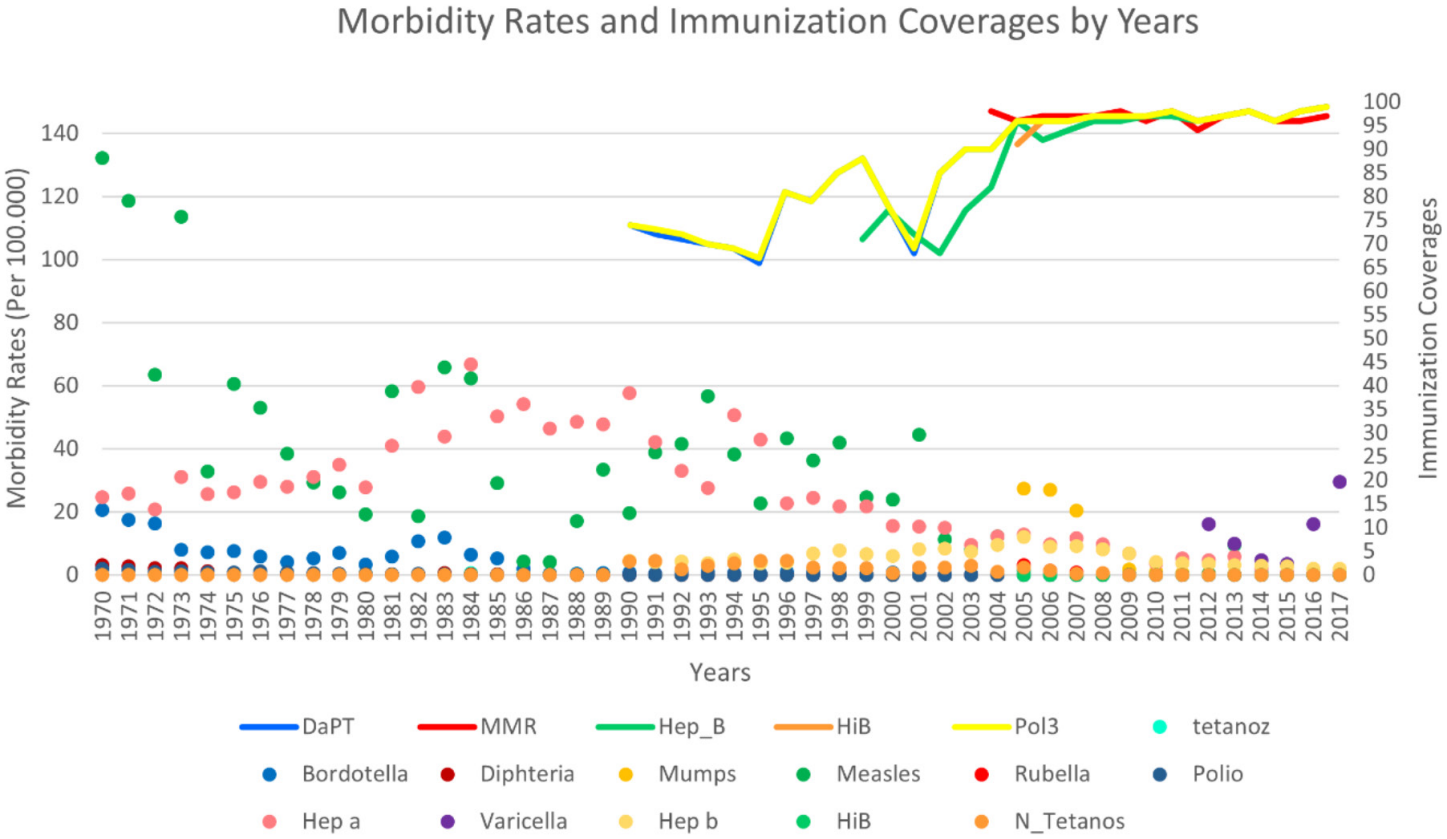
The comparision of morbidity rates (per 100.000) and vaccine coverages by years]. The line chart shows vaccine coverages, and as it is shown with numbers on the right side, it increases from 0 to 100 from 1970 to 2020. But immunization records usually started at 1990, the lines start at 1990. DaPT line represents Diphteria, Pertussiss, and Tetanus; MMR line represents Mumbs, Measles, and Rubella; Pol3 represents Polio; Hep_B represents Hepatitis B; Hib represents H.influenza type b. The dot plot in the figure shows morbidity rates. As it can be seen by the numbers on the left side, the mortality rate increases from 0 to 125 (per 100.000), when the timeline goes from 1970 to 2020.

The fact that the registration system for different diseases was initiated in different dates and in turn, the related graphics were enabled only after the launch of registration can be considered the limitation of this study. Also, there is no full confidence behind the vaccine records kept on papers used before the electronic registration system.

## 3. Results

The evaluation of vaccination activities, the number of cases and deaths in the country, and the years when vaccines were introduced into the routine vaccination schedule are presented in Table. Diphtheria and pertussis vaccine came into use in 1937, then the Polio vaccine was started to be used in 1963 with the attenuated form. After the start of the Polio eradication program in 1989, polio national vaccination days were implemented in 1995 and 1996. Measles immunization started in 1970 and the measles elimination program has been maintained since 2002. With the start of the measles elimination program, Measles school vaccination days (2003) and Measles vaccination days (2005) were organized. Hib, Mumps, and Rubella were added to the routine vaccination schedule in 2006. Hep B immunization started in 1998 and Hep A started in 2012 (Table).

**Table:** National immunization schedule performed in Turkey [2].

1937 Diphteria, Pertussis Immunization
1952 BCG Immunization
1963 Live Polio Immunization
1968 DTP Immunization
1970 Measles Immunization
1981 Expanded Programme on Immunization
1985 Vaccine Campaign in Turkey
1989 Poli Eradication Programme
1995 Polio National Immunization Days
1996 Measles Vaccination Acceleration Campaign
1997 Polio Mop‐Up
1998 Last Polio Case and Hepatitis B İmmunization
2003 Vaccination Days at Schools for Measles
2004 Initiation of Td vaccine for adults in necessary situations
2005 Measles Vaccination Days
2006 Addition of Hib, Mumps, Rubella Vaccines to Vaccination Schedule, Initiation of Hepatitis B Immunization for Adolescents
2007 Completion of Hepatitis B and Rubella Vaccines in Elementary students
2008 Administration of Pentavalent (DTP-Polio and Hib) Vaccine
2008 Inclusion of a Heptavalent Pneumococcal Conjugate Vaccine to Vaccination Schedule
2009 Elimination of Maternal-Neonatal Tetanus
2010 Initiation of DTP and IPV vaccines instead of live polio and Td vaccines at 1. Grade of Elementary School
2011 Initiation of 13-Valent Pneumococcal Conjugate Vaccine
2012 Hepatitis A Immunization
2013 Adding Chickenpox Vaccine to Current Vaccination Schedule

While the mortality rate (per 100.000) of pertussis was 0.59, it decreased below 0.1 after immunization. The mortality rate of diphtheria was nearly 2 in 1970 and is always under 0.01 after 1990. When neonatal tetanus was recorded in the 1990s, the mortality rate was 16.9 and showed fluctiations until 2006. In 2004, the Td vaccine application was started for adult vaccination and in 2009, the maternal-neonatal tetanus elimination program was started (Table). In 2010, the morbidity rate decreased to 0.16 and the mortality rate to 1.55 and did not increase again. On the other hand, considering the country vaccination status, there was a decrease in DaPT coverage, also an increase in neonatal tetanus deaths towards 1995. DaPT coverages have increased since 2003 and neonatal tetanus progressively decreased. While the Polio mortality rate was 1.1 in 1970, the number of cases and deaths decreased rapidly with vaccination campaigns and the last case was seen in 1998. The measles mortality has decreased to zero before 1990s with the effect of vaccinations that started to be used in 1970. Mumps vaccination was added in 2006 and coverages were always at high levels. Mumps morbidity rate was over 25 in 2006 and became almost zero in a few years (Figure 2).

The increase of pertussis immunization decreases the morbidity. The morbidity rate (per 100.000), which was 20.58 in 1970, decreased to 0.8 in 1990. The strong decline in vaccination in 1995 seems to somewhat negatively affect morbidity. The diphtheria morbidity rate was almost 3 in 1970 and it was under 0.01 after 2000. The immunization records of measles begin in 2006, coverages are always 94 and mostly over 96. Measles morbidity rates were stable after 2004 and are very low in the chart (Figure 2). The rubella morbidity rate was 3.12 in 2005. Rubella vaccination begins in 2006, and the morbidity rate in 2006 was 1.45, which dropped drastically over the years. The morbidity rate for hepatitis B peaked and reached almost 12 in 2005. Immunization started in 2006 and was generally at high levels. A moderate decrease in mortality rates is also observed after vaccination; however, associating this fact to vaccination is difficult. Since hepatitis A vaccination records were not available, they were not included in the vaccination chart. Hepatitis B morbidity, on the other hand, performed the last peak before vaccination in 2005 and decreased from 12 to 2 in 2017 (Figure 2).

The comparision of mortality rates (per 100.00) and vaccine coverages by years are given in Figure 1. The comparision of morbidity rates (per 100.000) and vaccine coverages by years are given in Figure 2.

## 4. Discussion

In general, the debate on the necessity and side effects of vaccines existed in human history for more than 200 years still continues today, so elaboration is very important to show the change in mortality and morbidity of communicable diseases with the introduction of vaccination.

In previous years, there may be doubts about the trustworthy of the records due to reasons such as not keeping the records of vaccination and cases or deaths electronically and meticulously as they are now, and not tracking vaccines with barcodes. However, it is assumed that the morbidity and mortality rates in those years were not less than the ones presented in this study; instead, they may be higher than given. While the recording systems have been gradually developed, the numbers have decreased and sometimes increased due to various reasons. To make it easier to understand, vaccination activities and changes in case numbers are presented by years as well as the examples across the world and comparisons respectively. Lastly, related debates took place in discussion section for each vaccine starting from the years when they were first introduced in the country.

### 4.1. Diphtheria- Pertussis -Tetanus- Neonatal Tetanus

Diphtheria and pertussis vaccine was applied for the first time in 1937 in Turkey (Table). While the mortality rate of pertussis cases was 0.59 in 1970, it declined rapidly to under 0.1 after the beginning of the DaPT immunization. The morbidity rate which was 20.58 in 1970 became 0.50 after 1990; however, the effect of a strong spike to bottom of immunization coverages in 1995 made pertussis morbidities visible again in mild degree over of the zero line in Figure 2. The pertussis morbidity rate was over 1 in 1996 and 1997.

As the diphteria morbidity rate is almost 3 in 1972, it is obvious on Figure 2. After 2000, together with other effects; it dropped under 0.01 and it has been going under 0.0001 thank to vaccination. The mortality rate was around 2 in 1970’s while it was under 0.01 just before 1990, in parallel to the success with diphteria has been ongoing since 1990 with the contribution of immunization, as well.

The first neonatal tetanus deaths were reported in 1990 and is visible in orange in Figure 1 and it was over 20 at the beginning of the recordings. In 2009, Maternal-Infant tetanus elimination program was implemented all around Turkey. Elimination program is still in force. Although tetanus cases, related morbidities and mortalities are just a few all over Turkey, small fluctuations can be seen from time to time.

After the effective vaccination campaigns, no newborn babies have died in Turkey due to neonatal tetanus since 2014. However, while tetanus morbidity had been decreasing until 2007 and the morbidity increased due to tetanus between 2008–2017. Adult vaccinations should be encouraged to protect infants in combating diseases such as tetanus that do not develop permanent immunity [6].

### 4.2. Poliomyelitis

The administration of Atennue Polio vaccine started in 1963 in Turkey. The mortality rates for Poliomyelitis in 1970 were 1.10. In 1989, Polio eradication program was started and Polio national vaccination days and Polio Mop-Up campaigns were performed in 1995 and 1997. After all this efforts, the last case was seen in 1998. (Table).

There is a risk in Turkey in terms of polio disease due to the migration after the civil war was broken out in Syria. To eliminate this risk, Turkey has started vaccinating all Syrian children and 5 million children under age 5 in 7 provinces before they enter the camps located near Syria. Turkey reached a vaccination rate of almost 90% with successful vaccination campaign. Thus, no polio cases have been reported and maybe possible child deaths have been prevented. However, the failure to implement strategic approaches causes the virus to continue to live and infect children. It is stated that if polio does not stop and spreads in Afghanistan, Nigeria, and Pakistan, where the virus is endemic, it can cause the emergence of 200,000 new cases annually worldwide World Health Organisation (2019). Poliyomyelitis factsheets [online] Website https://www.who.int/news-room/fact-sheets/detail/poliomyelitis [accessed 11.09.2020].. Consequently, it should be remembered that in the globalized world where transportation is now very easy, infectious diseases are considered as a problem that should be adressed not only by the endemic countries but the all countries in the world. The coverage has had a decent line since 2007 (Figure1 and 2).

### 4.3. Measles

Although measles vaccination application initiated in 1970, immunization coverage data is started to be kept since 2006. However, the immunization effect on measles can be easily understood by looking at the mortality chart (Figure 1), because there has been a linear decline since 1970 towards 1985 without immunization data. Looking at the morbidity, we can say that there is a stable status since 2004, because we clearly observe the impact of the measles elimination campaings in 2005 (Table and Figure 2). According to the vaccine coverage data started to be kept in 2006, this vaccine coverage, which is always over 94% and mostly 96% or above since this year, is the reason of this stable status.

Since 2002, the Ministry of Health has implemented the Measles Elimination Program. Measles vaccination days were organized at schools in 2003 and school-age children were immunized. Turkey has continued its commitment to the elimination of measles by implementing measles vaccination days for the second time in 2005. The significant impact of the second dose of vaccine, which was added in 1998 and administered to first-year children in school age, has been observed since 2001 (Table and Figure 2). From 2007 to 2011, the number of cases remained below 5, and no domestic cases were seen between 2008 and 2010. However, after the civil war broken out in 2011 in Syria and the measles outbreak in Europe because of suboptimum vaccination coverage, the morbidity rates started to increase rapidly and an epidemic was experienced in 2013 and the morbidity rates reached nearly 10 (there is an overlap between measles and varicella in 2013 on Figure 2) [7] (Figure 1 and 2). The increasing number of cases with strict vaccination campaigns could be prevented.

### 4.4. Haemophilus Influenzae Type B (Hib)

In 2006, 28 cases were observed in Turkey when vaccination was started, but no deaths were reported. Then, the number of cases decreased by years and no cases have been reported since 2013. In the United States, 8 cases were reported in 2015 and 27 cases under 5 were reported in Europe [8,9]. In some limited and hospital studies conducted in Turkey some Hib meningitis cases were diagnosed and according to the data provided from the Ministry, 55 cases were detected between 2006–2017. This fact reveals the need to support and continue the surveillance system in Turkey [10].

### 4.5. Mumps

In Turkey, the mumps vaccine was included in the vaccine calendar as triple monovalent, together with measles and rubella vaccines in 2006. There are not enough records about mortality rates before the vaccination in Turkey but just after the immunization in 2005 and 2006, morbidity rates became 27. It is clear that the rate was over the line of 25 on Figure 2 and it became almost invisible in several years. An analysis of the data provided from the whole country reveals that there is no death.

In one study conducted in Germany, the incidence was found to be 10.3 per 100.000, while in another study the incidence before vaccination was estimated to be between 100–1000 [11,12]. As presented by Anderson et. al., the incidence of mumps significantly decreased after vaccination campaigns were started. In the USA, a 97% decline was observed between 1968–1982. After vaccination practices, the annual incidence in Finland is 1 per 100,000 [11].

Vaccination is still important for this disease, which can still cause epidemics from time to time in the world. According to the 2005 report, 110 out of the 193 Member States of the WHO added the mumps vaccine into their national vaccination programmes [13].

### 4.6. Rubella

There are not enough records about mortality rates for rubella before the application of vaccination in Turkey, but after the initiation of the immunization in 2005 and 2006, morbidity rates were 3.12 and 1.45, respectively. In addition, it is over the line of 0 on Figure 2, and later, it is marked on the line of 0 due to the initiating of Rubella elimination programme. However, if we look at in Africa and South-East Asia where the vaccination practices are at the lowest level, still many congenital Rubella syndromes are reported [14].

In the light of this information, it is obvious that the only way to contain this virus, which causes many congenital anomalies which are irreversible or requiring high treatment costs, is vaccination and it should be applied rapidly in a serious manner by the other countries, while the necessity of continuation of the current vaccination activities is obvious in Turkey.

### 4.7. Hepatitis B

Hepatitis B immunization schedule launched in 1998 (Table). Since Hepatitis B mortalities are always below 1, it is difficult to distinguish an improvement in the Figure 1. Also, it is difficult to comment about that chronic disease, since its morbidity level is affected by different positive factors. However, morbidity decreased moderately after peaking in 2005, from 12 in 2005 to 2 in 2017. The fact that the coverage of Hepatitis B vaccine has increased by 96% from 2007 till today contributes a lot to this success factors (Figure 1).

There are relatively different HBsAg positivity rates in Turkey, which results from socio-cultural differences between provinces. For example, while HBsAg positivity was found as 12.5% in children between 2 months and 15 years of age in a study conducted in one part of Turkey, it was found as 1.7% in children between newborns and the ones aged 18 in a study conducted in Erzurum in 2004, and found as 9.5% in another study conducted in Van in 2005. The number of new hepatitis B cases in 2017 was 1571 in Turkey. The seropositivity rate and the number of cases indicate that studies on this subject should continue to be carried out in the same determination level for addressing the coverage of the vaccine [15,16,17].

### 4.8. Hepatitis A

Hepatitis A immunization launched in 2012 (Table). The vaccine coverage showing Hepatitis A vaccination is not available in the WHO database. Therefore, there is no line belonging to Hepatitis A in the figures indicating the immunization coverage. Hepatitis A morbidity rate decreases moderately after having a peak in 2005, from 12.8 in 2005 to 0.58 in 2017. Mortality rate was almost 2 in 1970; however the mortality rates dropped below 0.1 after vaccination in 2012 (Figure 1). Undoubtedly, morbidity rates provide a better understanding of the condition of the disease in society. The morbidity rates which were almost 5 in 2012 and 2013 is now decreasing and still under 1 thanks to the immunization.

### 4.9. Varicella

The coverage of varicella vaccination is not available in the WHO database. Therefore, no line belonging to varicella is marked in the figures. Varicella diagnosis leads some confusion. A study conducted about cost-effectiveness analysis of universal varicella vaccination in Turkey shows age-specific reductions in varicella incidence and also indicates that incidence under 15 years old is 2.39 while it is mostly more than 10 for all age groups over 55. This distribution makes it difficult for us to talk about the effect of vaccine coverage on morbidity [18].

The study shows that the relationship between vaccine coverages, mortality and morbidity rates for example: how morbidity rates of pertussis increased in 1995 when pertussis immunization coverages decreased?, how mortality rates of neonatal tetanus increased when tetanus coverage reduced in 1995?, and how neonatal tetanus mortality decreased when coverages of tetanus immünization increased in 2003?. Poliomyelitis immunization has been at a decent level since 2007 in the figures represented. The fact that how measles days reduced measles morbidity has been shown. The effect of vaccination on mumps morbidity gives a hint about the effects of vaccination.

As a result, the data indicating how diseases and disabilities are prevented and how the lives are saved through immunization is presented by looking at the country’s history. We can say that many possible outbreaks have been prevented by vaccination studies by now and the outbreaks have been overcome through tightly conducted vaccination campaigns, though occassional cases have occured. In this regard, the current study demonstrating the importance of vaccination claims that all individuals in the society have a responsibility in this scope when the communicable diseases and tackling infectious diseases are the cases. The main responsibility is to ensure that they and their children are vaccinated against the communicable diseases which are risky for society’s health.
